# Smart meta-superconductor MgB_2_ constructed by the dopant phase of luminescent nanocomposite

**DOI:** 10.1038/s41598-019-50663-6

**Published:** 2019-10-02

**Authors:** Yongbo Li, Honggang Chen, Mingzhong Wang, Longxuan Xu, Xiaopeng Zhao

**Affiliations:** Smart Materials Laboratory, Department of Applied Physics, Northwestern Polytechnical University, Xi’an, 710072 China

**Keywords:** Superconducting properties and materials, Superconducting properties and materials

## Abstract

On the basis of the idea that the injecting energy will improve the conditions for the formation of Cooper pairs, a smart meta-superconductor (SMSC) was prepared by doping luminescent nanocomposite Y_2_O_3_:Eu^3+^/Ag in MgB_2_. To improve the superconducting transition temperature (*T*_*C*_) of the MgB_2_-based superconductor, two types of Y_2_O_3_:Eu^3+^/Ag, which has the strong luminescence characteristic, with different sizes were prepared and marked as m-Y_2_O_3_:Eu^3+^/Ag and n-Y_2_O_3_:Eu^3+^/Ag. MgB_2_ SMSC was prepared through an *ex situ* process. Results show that when the dopant content was fixed at 2.0 wt.%, the *T*_*C*_ of MgB_2_ SMSC increased initially then decreased with the increase in the Ag content in the dopant. When the Ag content is 5%, the *T*_*C*_ of MgB_2_ SMSC was 37.2–38.0 K, which was similar to that of pure MgB_2_. Meanwhile, the *T*_*C*_ of MgB_2_ SMSC doped with n-Y_2_O_3_:Eu^3+^/Ag increased initially then decreased basically with the increase in the content of n-Y_2_O_3_:Eu^3+^/Ag, in which the Ag content is fixed at 5%. The *T*_*C*_ of MgB_2_ SMSC doped with 0.5 wt.% n-Y_2_O_3_:Eu^3+^/Ag was 37.6–38.4 K, which was 0.4 K higher than that of pure MgB_2_. It is thought that the doping luminescent nanocomposite into the superconductor is a new means to improve the *T*_*C*_ of SMSC.

## Introduction

Improving the superconducting critical transition temperature of materials is an important scientific and technical problem in condensed matter physics and materials science. Recently, Fausti *et al*.^1^ used mid-infrared femtosecond pulses to transform non-superconducting La_1.675_Eu_0.2_Sr_0.125_CuO_4_ into a transient 3D superconductor. A similar method was also applied to investigate YBa_2_Cu_3_O_6.5_^2^ and K_3_C_60_^3,4^ and good experimental results were achieved. Ye *et al*. have reported the observation of field-induced superconductivity of ZrNCl and MoS_2_ by quasi-continuous electrostatic carrier doping achieved by combining liquid and solid gating^5,6^. Drozdov *et al*.^7^ reported conventional superconductivity at 203 K under high pressure in a sulfur hydride system. Adu *et al*.^8^ increased the T_C_ of commercial “dirty” MgB_2_ by conducting non-substitutional hole-doping of the MgB_2_ structure using minute, single-wall carbon nanotube inclusions. In accordance with homogeneous system theory^9^, Smolyaninov *et al*.^10–12^ stated that a superconducting metamaterial with an effective dielectric response function that is less and approximately equal to zero may exhibit high T_C_, and they verified this theory in their subsequent experiments. Recently, Cao *et al*.^13,14^ investigated correlated insulator behavior at half-filling in magic-angle graphene superlattices and reported the realization of intrinsic unconventional superconductivity in a 2D superlattice created by stacking two sheets of graphene that are twisted relative to each other at a small angle. Another important method for studying superconductivity is the topological superconductors^15–20^, which have attracted great attention in condensed matter physics. However, obtaining a practical superconductor with high *T*_*C*_ remains difficult.

The superconductivity of MgB_2_ was discovered in 2001^21^. MgB_2_ is a promising material with large-scale applications because of its excellent superconducting properties and simple crystal structure^22–27^. Considering that the *T*_*C*_ of MgB_2_ is close to the McMillan temperature limit^28,29^, developing an effective experimental method to improve the *T*_*C*_ of MgB_2_ is beneficial to its practical application and to the understanding of the superconducting mechanism. Chemical doping is a simple, effective, commonly used method to change the *T*_*C*_ of superconducting materials. However, many experimental results have confirmed that conventional chemical doping decreases the *T*_*C*_ of MgB_2_^30–36^. To date, no effective method has been developed to improve the *T*_*C*_ of MgB_2_. The use of metamaterial structures to achieve special properties is an important method developed in recent decades^37–41^, and it provides a new approach to improve the *T*_*C*_ of superconducting materials.

On the basis of metamaterials, our group investigated the effects of ZnO electroluminescent (EL) material doping on the superconductivity of BSCCO in 2007 and attempted to change the *T*_*C*_ of this superconductor^42^. Meanwhile, it is proposed that the combination of chemical doping and EL excitement, that is, doping EL materials in superconducting materials to form a meta structure, may be an effective method to improve the *T*_*C*_ of superconductors^43^. On the basis of these results, a smart meta-superconductor (SMSC) model for improving the *T*_*C*_ of materials has been proposed recently. In the model, the dopant phase is used to inject energy through its EL under the external field to strengthen the Cooper pairs, thereby achieving the purpose of changing the *T*_*C*_. Zhang *et al*.^44^ prepared MgB_2_ doped with Y_2_O_3_:Eu^3+^ particles through an *in situ* process. Tao *et al*.^45^ prepared MgB_2_ doped with Y_2_O_3_:Eu^3+^ nanorods with different EL intensities through an *ex situ* process. Their results indicated that doping EL materials is favorable for the improvement of *T*_*C*_ compared with conventional doping, which always reduces the superconducting transition temperature of the sample. In addition, similar experimental results were obtained by replacing Y_2_O_3_:Eu^3+^ with Y_3_VO_4_:Eu^3+^ flakes^46^. Meanwhlie, results also indicated that the *T*_*C*_ can be changed by adjusting the Y_2_O_3_:Eu^3+^ concentration and EL exciting current^47^. However, there are some problems need to improve in the experiments. Y_2_O_3_:Eu^3+^ particles or flakes tended to agglomerate during the preparation process and its EL intensity was weak^44–47^. The quality of commercial MgB_2_ was poor and its superconducting transition width (*ΔT*) is 4.6 K^46,47^, further experiment and improvement will be needed. Moreover, the dopants would decrease the *T*_*C*_ in the case of double dopants, i.e., simultaneous incorporation of Y_2_O_3_:Eu^3+^ and nano-Ag into MgB_2_ ^47^.

In this paper, a kind of new phase of luminescent nanocomposite Y_2_O_3_:Eu^3+^/Ag was prepared by compounding nano Ag into the Y_2_O_3_:Eu^3+^ matrix directly^48^. The luminescent intensity of Y_2_O_3_:Eu^3+^/Ag is three times higher than that of Y_2_O_3_:Eu^3+^ due to the composite illumination of EL and photoluminescence (PL). Two kinds of nanocomposite illuminator Y_2_O_3_:Eu^3+^/Ag with different sizes, namely, micro Y_2_O_3_:Eu^3+^/Ag (m-Y_2_O_3_:Eu^3+^/Ag) and nano Y_2_O_3_:Eu^3+^/Ag flakes (n-Y_2_O_3_:Eu^3+^/Ag), are prepared. Meanwhile, a new kind of commercial MgB_2_ with a small *ΔT* of 0.8 K was used. SMSC was prepared by doping Y_2_O_3_:Eu^3+^/Ag in MgB_2_ through an *ex situ* process^49^. The *T*_*C*_ of the MgB_2_-based superconductor is investigated by changing the Ag content in the dopant phase, the sizes of the dopant phase, and the doping concentration. The results indicate that the *T*_*C*_ of MgB_2_ doped with 0.5 wt.% n-Y_2_O_3_:Eu^3+^/Ag is 37.6–38.4 K, which is 0.4 K higher than that of pure MgB_2_, which further confirmed that the SMSC is a new way to improve the critical transition temperatures.

## Experiment

### Preparation of m-Y_2_O_3_:Eu^3+^/Ag and n-Y_2_O_3_:Eu^3+^/Ag

Y_2_O_3_ and Eu_2_O_3_ were weighed (the atomic ratio of Y and Eu is 0.95:0.05) and dissolved in a beaker with excess concentrated hydrochloric acid and subsequently heated and dried at 70 °C for 2 h to obtain a white precursor. One of the precursor was dissolved in 4 mL of deionized water to form a solution, and ammonium oxalate was added to it dropwise. The solution was subsequently stirred vigorously at 2 °C in a temperature-controlled water bath. A certain amount of AgNO_3_ was added to the solution after been stirred for 30 min. After another 30 min of stirring, the pH value of the solution was adjusted to 9–10 by adding NaOH. The final solution, designated as solution A, was obtained after another 30 min of stirring. Another precursor was also prepared into solution with 24 mL benzyl alcohol. Octylamine (4 mL) was added dropwise to the solution, which was subsequently stirred for 1 h. Afterward, a certain amount of AgNO_3_ was added to the solution. After stirring for another hour, another solution was obtained and designated as solution B. Solutions A and B were then transferred to two reaction kettles, respectively. A hydrothermal reaction occurred at 160 °C for 24 h. The products were washed several times with deionized water and absolute ethanol and sintered at 800 °C for 2 h to form Y_2_O_3_:Eu^3+^/AgCl. After illumination, the Y_2_O_3_:Eu^3+^/AgCl transformed into two kinds of luminescent Y_2_O_3_:Eu^3+^/Ag nanocomposite with different sizes and a certain amount of Ag. The two luminescent nanocomposite materials were designated as m-Y_2_O_3_:Eu^3+^/Ag and n-Y_2_O_3_:Eu^3+^/Ag. Y_2_O_3_:Eu^3+^/Ag with different Ag contents was prepared by changing the AgNO_3_ content. In this paper, Ag contents uniformly refers to the initial nominal atomic ratio of Ag and Y. For example, 5% Ag means that the initial nominal atomic ratio of Ag to Y is 0.05:0.95. Meanwhile, similar method was applied to synthesize Y_2_O_3_ and Y_2_O_3_:Sm^3+^.

### Preparation of MgB_2_–based SMSC

At a certain ratio, commercial MgB_2_ powder (Alfa Aesar) and the luminescent nanocomposite Y_2_O_3_:Eu^3+^/Ag were weighed and prepared into an alcohol solution. The two suspensions were sonicated for 20 min, then the dopant was added dropwise to MgB_2_. After sonication for more than 20 min, the mixed solution was transferred to a culture dish. Subsequently, the culture dish was placed in a vacuum oven at 60 °C for 4 h to yield a black powder. The powder was pressed into a pellet with a diameter of 11 mm and a thickness of 2 mm and placed in a small tantalum container, which was annealed at 800 °C for 2 h in high-purity argon atmosphere. The MgB_2_-based superconductor doped with luminescent nanocomposite materials of different sizes and Ag contents was synthesized to investigative the *T*_*C*_ of SMSC.

## Results and Discussion

**Figure 1:**
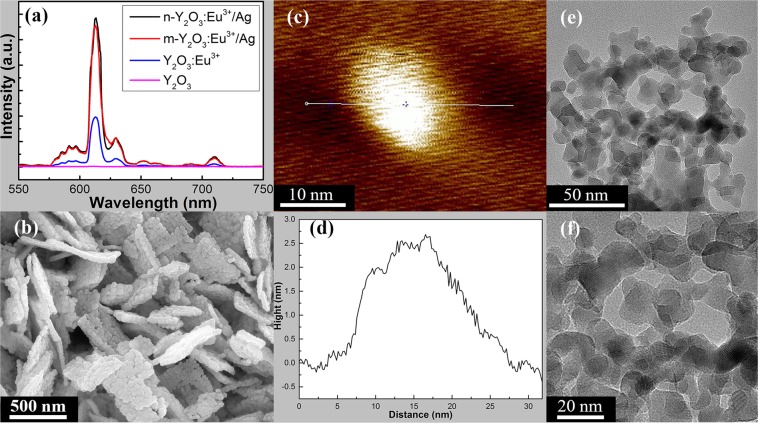
(**a**) EL spectra of Y_2_O_3_, Y_2_O_3_:Eu^3+^, m-Y_2_O_3_:Eu^3+^/Ag, and n-Y_2_O_3_:Eu^3+^/Ag; (**b**) SEM image of m-Y_2_O_3_:Eu^3+^/Ag; (**c**,**d**) AFM images and (**e,f**) TEM images of n-Y_2_O_3_:Eu^3+^/Ag.

Figure 1a shows the EL spectra of Y_2_O_3_, Y_2_O_3_:Eu^3+^, m-Y_2_O_3_:Eu^3+^/Ag, and n-Y_2_O_3_:Eu^3+^/Ag. The Ag content of the luminescent nanocomposite materials was 5.0%. It shows that Y_2_O_3_ is a non-EL material and becomes a kind of EL material after the addition of a small amount of Eu element. The results also indicate that the EL intensity of the luminescent m-Y_2_O_3_:Eu^3+^/Ag nanocomposite and n-Y_2_O_3_:Eu^3+^/Ag is remarkably improved primarily due to the composite luminescence of the electroluminescence of Eu^3+^ centric and the surface plasma-enhanced photoluminescence of Ag. Among the four dopants, n-Y_2_O_3_:Eu^3+^/Ag had the highest EL intensity. Figure 1b shows the SEM image of m-Y_2_O_3_:Eu^3+^/Ag. The surface size and thickness of the m-Y_2_O_3_:Eu^3+^/Ag flake are approximately 300 nm and 30 nm, respectively. Figure 1c,d show AFM images of n-Y_2_O_3_:Eu^3+^/Ag and Fig. 1d presents a cross section of the AFM image in Fig. 1c. Figure 1e,f show TEM images of n-Y_2_O_3_:Eu^3+^/Ag. It can be seen that the surface size of n-Y_2_O_3_:Eu^3+^/Ag was 20 nm, and its thickness was approximately 2.5 nm, which is much smaller than that of m-Y_2_O_3_:Eu^3+^/Ag.

**Figure 2:**
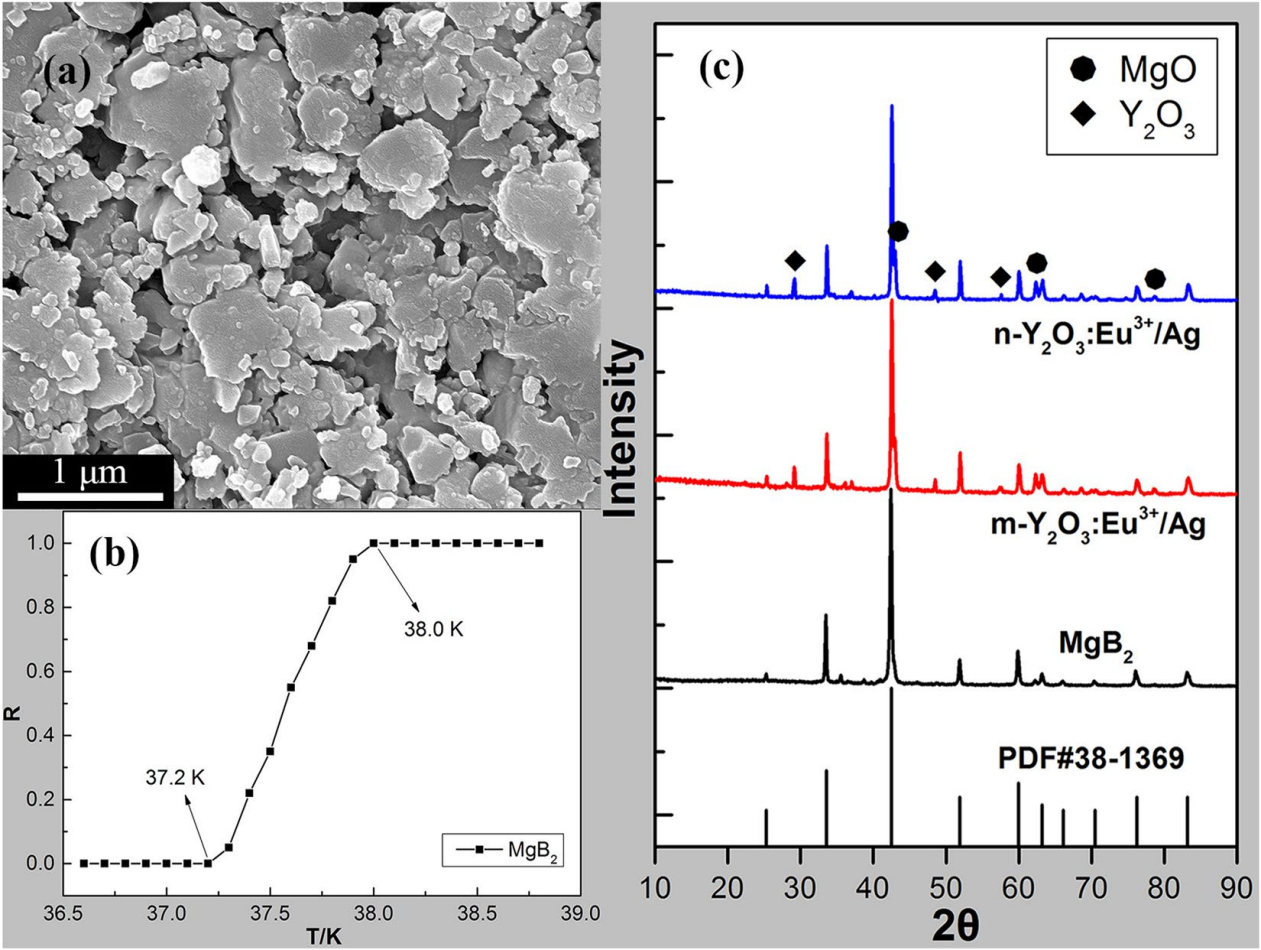
(**a**) SEM image and (**b**) normalized temperature-dependent resistivity (*R–T*) curve of pure MgB_2_; (**c**) XRD spectra of pure MgB_2_ and MgB_2_ doped with 2.0 wt.% m-Y_2_O_3_:Eu^3+^/Ag and 2.0 wt.% n-Y_2_O_3_:Eu^3+^/Ag.

Figure 2a shows the SEM image of pure MgB_2_. The size of the MgB_2_ particle was approximately 0.1–1 μm. The *T*_*C*_ of the samples was determined based on the *R–T* curve, which was measured using a four-probe method in a liquid helium cryogenic system developed by the Advanced Research Systems Company. Figure 2b shows the normalized *R–T* curve of pure MgB_2_ and indicates that the onset temperature (*T*_*on c*_) and offset temperature (*T*_*off c*_)^50,51^ of pure MgB_2_ were 38.0 and 37.2 K, respectively. The *ΔT* of pure MgB_2_ was 0.8 K. Figure 2c shows the XRD spectra of pure MgB_2_ and partially doped samples, in which the standard card of MgB_2_ (PDF#38-1369) is demonstrated using black vertical lines. The results showed that the XRD spectrum of pure MgB_2_ (black curve) matched the standard card of MgB_2_ well, except for the inevitable small amount of the MgO phase^52–55^. The red and blue curves represent the XRD spectra of MgB_2_ doped with 2.0 wt.% m-Y_2_O_3_:Eu^3+^/Ag and 2.0 wt.% n-Y_2_O_3_:Eu^3+^/Ag, respectively. The Ag content was 5.0%. The main phase of the doped samples was MgB_2_. Moreover, apart from a small amount of the MgO phase, the Y_2_O_3_ phase was also found in the XRD spectra of the doped samples. The XRD spectra of the other doped samples were similar.

**Figure 3:**
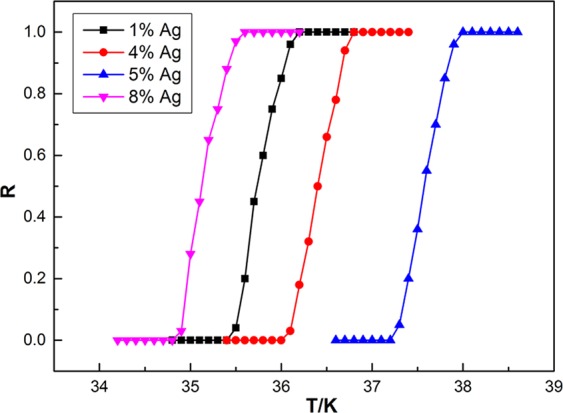
Normalized *R–T* curves of MgB_2_ doped with 2.0 wt.% m-Y_2_O_3_:Eu^3+^/Ag with different Ag contents.

Figure 3 shows the normalized *R–T* curves of MgB_2_ doped with m-Y_2_O_3_:Eu^3+^/Ag with different Ag contents. On the basis of the results of our previous study^45,46^, the content of m-Y_2_O_3_:Eu^3+^/Ag in the four samples was fixed at 2.0 wt.%. The Ag contents of m-Y_2_O_3_:Eu^3+^/Ag in the four samples were 1.0%, 4.0%, 5.0%, and 8.0%, as shown in the figure, and their *T*_*C*_ values were 34.8–35.6, 36.0–36.8, 37.2–38.0, and 34.8–35.6 K, respectively. The *T*_*C*_ of the doped samples initially increased then decreased with the increase in Ag content. Meanwhile, the corresponding doped sample had the highest *T*_*C*_ when the concentration of m-Y_2_O_3_:Eu^3+^/Ag was fixed at 2.0 wt.% and the Ag content was 5.0%, which is equal to that of pure MgB_2_. The experimental results are similar to those of our previous studies, that is, doping EL materials may improve *T*_*C*_ in several cases compared with conventional doping, which always reduces the *T*_*C*_ of the sample. As a dopant, m-Y_2_O_3_:Eu^3+^/Ag exerts an impurity effect that decreases *T*_*C*_. Meanwhile, as an EL material, m-Y_2_O_3_:Eu^3+^/Ag exerts an EL exciting effect that increases *T*_*C*_ ^45,46^. An obvious competitive relationship exists between the impurity effect and the EL exciting effect. The final *T*_*C*_ of the samples increased when the EL exciting effect was fully utilized and the impurity effect was minimized.

**Figure 4:**
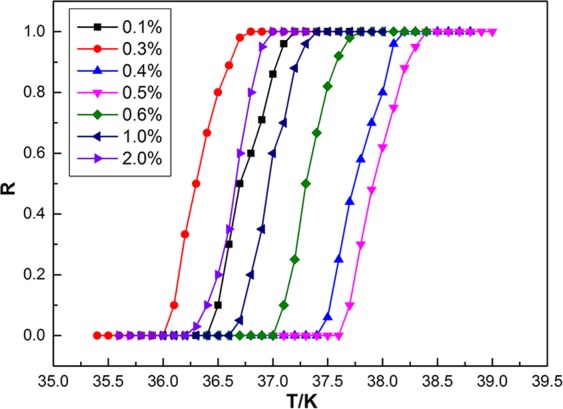
Normalized *R–T* curves of MgB_2_ doped with n-Y_2_O_3_:Eu^3+^/Ag.

Figure 4 shows the normalized *R–T* curves of MgB_2_ doped with 0.1–2.0 wt.% n-Y_2_O_3_:Eu^3+^/Ag. Ag concentration was fixed at 5.0%. It can be seen that *T*_*C*_ of MgB_2_ doped with n-Y_2_O_3_:Eu^3+^/Ag initially decreased, increased, then decreased again with the increase in doping concentration. A too low or too high doping concentration reduces *T*_*C*_, which is similar to the finding of our previous study. When the doping concentration was in a low range, *T*_*C*_ decreased with the increase in doping concentration due to the dominance of the impurity effect of the dopant, which is similar to the results of conventional doping. The EL exciting effect of the dopant dominated with the further increase in doping concentration, resulting in the increase in *T*_*C*_. The samples doped with 0.5 wt.% n-Y_2_O_3_:Eu^3+^/Ag had the highest *T*_*C*_ of 37.6–38.4 K, which is 0.4 K higher than that of pure MgB_2_. However, the impurity effect of the dopant dominated when the doping concentration increased to a high range, which led to a low *T*_*C*_. These results indicate that doping luminescent nanocomposite materials effectively adjusts and improves *T*_*C*_ at an appropriate doping concentration.

**Figure 5:**
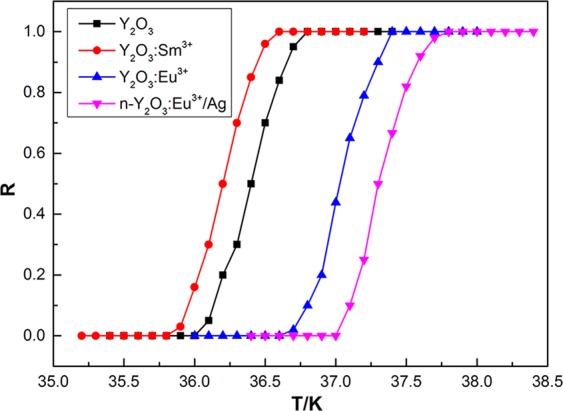
Normalized *R–T* curves of MgB_2_ doped with Y_2_O_3_, Y_2_O_3_:Sm^3+^, Y_2_O_3_:Eu^3+^, and n-Y_2_O_3_:Eu^3+^/Ag.

MgB_2_ doped with non-EL materials Y_2_O_3_ and Y_2_O_3_:Sm^3+^ were synthesized to prove the conclusions above. Figure 5 shows the normalized *R–T* curves of MgB_2_ doped with Y_2_O_3_, Y_2_O_3_:Sm^3+^, Y_2_O_3_:Eu^3+^, and n-Y_2_O_3_:Eu^3+^/Ag. The doping concentration was fixed at 0.6 wt.%, and the Ag content in n-Y_2_O_3_:Eu^3+^/Ag was 5.0%. Results indicated that *T*_*C*_ of MgB_2_ doped with non-EL materials Y_2_O_3_ or Y_2_O_3_:Sm^3+^ was much lower than that of pure MgB_2_, which is different from MgB_2_ doped with EL materials at the same concentration. MgB_2_ doped with Y_2_O_3_:Eu^3+^ increased to 36.6–37.4 K due to the EL exciting effect. Meanwhile, MgB_2_ doped with the luminescent n-Y_2_O_3_:Eu^3+^/Ag nanocomposite further increased to 37.0–37.8 K. The results show that doping EL materials facilitates an increase in *T*_*C*_ in several cases compared with conventional doping, which always reduces the *T*_*C*_ of the sample. Meanwhile, luminescent Y_2_O_3_:Eu^3+^/Ag nanocomposite materials increase the *T*_*C*_ of MgB_2_ due to the strong EL intensity.

The results in Fig. 4 show that the optimum concentration of n-Y_2_O_3_:Eu^3+^/Ag is 0.5 wt.%, which is lower than the value in our previous study^44–46^ due to the small size of n-Y_2_O_3_:Eu^3+^/Ag. The disadvantages caused by the impurity effect can be reduced if luminescent nanocomposite materials have a small size and are relatively evenly distributed in the sample. Moreover, the *ΔT* of commercial MgB_2_ in our previous study^46^ was too large to accurately determine the influence of the dopant phase on *T*_*C*_. In the current study, a new kind of commercial MgB_2_ with a small *ΔT* of 0.8 K was used, and we obtained a similar conclusion, which further proves the effectiveness of this method.

## Conclusions

In this paper, two types of luminescent nanocomposite Y_2_O_3_:Eu^3+^/Ag with different sizes were prepared and marked as m-Y_2_O_3_:Eu^3+^/Ag and n-Y_2_O_3_:Eu^3+^/Ag. SEM and AFM images indicated that the surface size and thickness of m-Y_2_O_3_:Eu^3+^/Ag are approximately 300 nm and 30 nm, which are 20 nm and 2.5 nm for n-Y_2_O_3_:Eu^3+^/Ag. The EL spectra showed that the luminescent intensity of Y_2_O_3_:Eu^3+^/Ag is three times higher than that of Y_2_O_3_:Eu^3+^. MgB_2_ of SMSC was prepared through an *ex situ* process to improve the *T*_*C*_ of the MgB_2_-based superconductor on the basis of the idea that the injecting energy will improve the conditions for the formation of Cooper pairs. The *T*_*C*_ of MgB_2_ SMSC was determined based on the measured *R–T* curve by using the four-probe method in a liquid helium cryogenic system. Results show that the *T*_*C*_ of MgB_2_ SMSC initially increased then decreased with the increase in the Ag content when m-Y_2_O_3_:Eu^3+^/Ag content was fixed at 2.0 wt.%. When the Ag content was 5.0%, the *T*_*C*_ of MgB_2_ SMSC doped with 2.0 wt.% m-Y_2_O_3_:Eu^3+^/Ag was 37.2–38.0 K, which was equal to that of pure MgB_2_. Meanwhile, the *T*_*C*_ of MgB_2_ SMSC doped with n-Y_2_O_3_:Eu^3+^/Ag increased initially then decreased basically with the increase in the content of n-Y_2_O_3_:Eu^3+^/Ag, in which the Ag content is fixed at 5%. The *T*_*C*_ of MgB_2_ SMSC doped with 0.5 wt.% n-Y_2_O_3_:Eu^3+^/Ag was 37.6–38.4 K, which was 0.4 K higher than that of pure MgB_2_. It is thought that the doping luminescent nanocomposite into the superconductor is a new means to improve the *T*_*C*_ of SMSC. However, the increase in the *T*_*C*_ remains insufficient. In future work, new dopant phases with improved characteristics will be prepared to increase the *T*_*C*_ of MgB_2_. Meanwhile, we will attempt to apply this method to other superconductors.
